# AI-BASED Tool to Estimate Sodium Intake in STAGE 3 to 5 CKD Patients—The UniverSel Study

**DOI:** 10.3390/nu17213398

**Published:** 2025-10-29

**Authors:** Maelys Granal, Nans Florens, Milo Younes, Denis Fouque, Laetitia Koppe, Emmanuelle Vidal-Petiot, Béatrice Duly-Bouhanick, Sandrine Cartelier, Florence Sens, Jean-Pierre Fauvel

**Affiliations:** 1 Hospices Civils de Lyon, Hôpital Edouard Herriot, Service de Néphrologie, Université Claude Bernard Lyon 1, 69003 Lyon, France; 2 Laboratoire de Biométrie et Biologie Evolutive, Equipe EMET Equipe Evaluation et Modélisation des Effets Thérapeutiques, Université Claude Bernard Lyon 1, 69100 Villeurbanne, France; 3 Department of Nephrology, Hôpitaux Universitaires de Strasbourg, 1 Place de l’Hôpital, 67000 Strasbourg, France; 4 Hospices Civils de Lyon, Centre Hospitalier Lyon Sud, Université de Lyon, Service de Néphrologie, Carmen, 69495 Pierre-Bénite, France; 5 CarMeN Laboratory, INSERM 1060, INRAE, Claude Bernard Lyon 1 University, 69310 Pierre-Bénite, France; 6 Physiology Department, ESH Hypertension Excellence Centre Bichat Hospital, AP-HP, Université Paris Cité, Inserm U1148, LVTS, 75018 Paris, France; 7 Department of Hypertension and Therapeutics, CHU Rangueil, 31400 Toulouse, France; 8 Centre AURAL—Lyon, 124 Rue Villon, 69008 Lyon, France

**Keywords:** Bayesian network, chronic kidney disease, epidemiology, machine learning, prediction tool, sodium diet

## Abstract

**Background**: Arterial hypertension is highly prevalent among patients with chronic kidney disease (CKD), acting both as a cause and consequence of declining kidney function, and significantly increasing cardiovascular risk. Among modifiable risk factors, diet—particularly excessive sodium intake—plays a central role in the prevention and personalized management of CKD. **Methods**: This study aimed to develop an innovative, digitally accessible tool to estimate sodium intake in stages 3 to 5 CKD patients, using 24-h urinary sodium excretion as the reference standard. **Results**: Twenty-five clinical, biological, therapeutic, and dietary variables were collected from 493 patients followed across 6 French centers. A probabilistic Tree-Augmented Naive Bayes model was used to develop the tool based on the 15 most informative variables. The model demonstrated an internal accuracy of 71%, indicating that predicted and observed sodium intake categories matched in 71% of cases. **Conclusions**: This AI-based prediction model offers a promising clinical tool to estimate daily sodium intake in patients with stages 3 to 5 CKD. However, external validation using independent national and international datasets is essential to establish its robustness and generalizability prior to implementation in routine clinical practice.

## 1. Introduction

Patients with chronic kidney disease (CKD) very frequently present with arterial hypertension (HTN), which acts both as a cause and a consequence of the progressive decline in kidney function. The prevalence of HTN increases with the severity of CKD [1,2] ranging from 60% to over 90%, depending on the disease stage and underlying etiology [2,3]. HTN is associated with a wide range of complications, including an elevated risk of stroke, ischemic heart disease, peripheral artery disease, CKD progression, retinopathies, and neurodegenerative disorders such as Alzheimer’s disease [4]. These adverse effects are primarily linked to arterial wall thickening, loss of elasticity, and the progression of atherosclerotic lesions in major arteries (e.g., carotid, coronary, renal, and femoral arteries) resulting from chronically elevated blood pressure.

Hypertension is influenced by numerous risk factors, some of which are non-modifiable (e.g., age, sex, family history, and CKD), while others are modifiable and largely related to lifestyle. The latter include smoking, physical inactivity, obesity, excessive alcohol intake, and an unbalanced diet—characterized by high sodium intake and insufficient potassium consumption [4]. Diet plays not only a preventive role in CKD, but is also a therapeutic tool that plays a central role in a personalized, and holistic approach to care [4,5]. On a global scale, salt intake remains well above recommended levels. In 2023, the World Health Organization (WHO) estimated the global average salt intake to be 10.8 g/day—more than twice the recommended limit of 5 g/day [6].

In this context, there is a critical need for reliable, user-friendly tools tailored to clinical practice, enabling healthcare professionals to accurately assess sodium intake. Such tools could support dietary interventions, optimize treatment strategies, and help prevent complications—particularly cardiovascular events—associated with inappropriate sodium consumption [7,8,9].

However, estimating sodium intake remains a methodological challenge. Commonly used tools, such as food frequency questionnaires (FFQs), 24-h dietary recalls, and food diaries, rely on patient self-reporting and are thus prone to recall bias, underreporting, and simplification. These limitations become more pronounced as the duration of data collection increases, especially beyond four days [10]. The current reference method for evaluating sodium intake is based on 24-h urine collection. Although more accurate, this method is burdensome, poorly suited for routine clinical use, and susceptible to collection errors [11,12]. To address this issue, an online self-assessment tool, ExSel was developed using data from 100 hypertensive patients, with 24-h urinary sodium excretion as the reference. While promising, this tool has not shown sufficient validity in patients with CKD. Another Canadian online tool designed to estimate sodium intake and provide personalized feedback has not been validated against 24-h urinary sodium excretion either [13,14].

The objective of our study was to develop an innovative, digitally accessible tool specifically designed to estimate sodium intake in patients with CKD. This tool was compared to the reference method of 24-h urinary sodium excretion, with sample quality verified by creatinine excretion measurement and patients interviews.

## 2. Materials and Methods

### 2.1. Study Design and Setting

The objective of this study is to develop a predictive tool for estimating sodium intake in patients with stage 3 to 5 chronic kidney disease (CKD), using data collected from six centers across France. This multicenter database is considered representative of the French population, owing to its geographic diversity and the broad spectrum of patient profiles. It includes data from university hospitals, medical associations, as well as both private and public healthcare institutions. Such diversity enabled the training of a machine learning model on a large and heterogeneous dataset. The study adheres to the TRIPOD reporting guidelines, as detailed in the Supplementary Materials (Table S1).

### 2.2. Participants

Data were prospectively collected from 1089 patients who underwent a 24-h urine collection as part of their routine clinical care. Eligible patients were those who attended one of the six participating centers between July 2023 and July 2024. The participating centers included:

1. Hôpital Lyon Sud—Hospices Civils de Lyon, Department of Nephrology;
2. AURAL Lyon—Association for the Use of Artificial Kidney in the Lyon Region;
3. Strasbourg University Hospitals, Department of Nephrology;
4. Toulouse University Hospitals—CHU Rangueil, Department of Hypertension and Therapeutics;
5. Assistance Publique—Hôpitaux de Paris, Bichat Claude Bernard Hospital, Department of Physiology and Functional Explorations;
6. Hôpital Edouard Herriot—Hospices Civils de Lyon, Department of Nephrology, Hypertension, and Dialysis.

Inclusion criteria were as follows:

- Male or female aged 18 years or older, not opposed to participating in the study;
- Outpatient or day-hospital patient in a nephrology or hypertension unit;
- Having completed a 24-h urine collection within one month prior to inclusion as part of routine care, including urinary sodium analysis.

The exclusion criteria were as follows:

(i) use of medications likely to interfere with 24-h natriuresis (e.g., sodium polystyrene sulfonate, locally acting antacids containing sodium, dietary supplements);
(ii) extrarenal sodium losses due to intense sweating, intensive physical exercise, vomiting, diarrhea, or breastfeeding;
(iii) individuals under legal protection (e.g., guardianship or curatorship).

### 2.3. Predictors and Outcome

Twenty-five variables were collected to estimate 24-h natriuresis, considered the reference for daily dietary sodium intake. These included patient characteristics (weight, height, age, sex, systolic blood pressure [SBP], and diastolic blood pressure [DBP]); comorbidities (type of kidney disease, presence of edema diagnosed during consultation, history of heart failure or nephrotic syndrome); biological data (serum creatinine, used to calculate estimated glomerular filtration rate [eGFR] via the CKD-EPI formula, and 24-h urinary sodium excretion); current treatments (antihypertensive and/or diuretic medications, including ARBs or ACE inhibitors, furosemide, thiazides, and spironolactone); and dietary responses from the UniverSel questionnaire.

Quantitative variables (age, weight, height) were discretized into five frequency-based classes. SBP was categorized into five clinically relevant classes: <120 mmHg, 120–129 mmHg, 130–139 mmHg, 140–159 mmHg, and >159 mmHg. DBP was categorized into four clinically relevant classes: <80 mmHg, 80–84 mmHg, 85–89 mmHg, and >89 mmHg. The type of CKD was classified into six categories: vascular, diabetic, tubulointerstitial, glomerular, autosomal dominant polycystic kidney disease (ADPKD), and other.

Additional variables were collected to describe the study population, including CKD-EPI to classify patients by CKD stage. Urine volume and urinary creatinine excretion were also recorded to verify the completeness of 24-h urine collection.

The database was cleaned, missing data were handled using Bayesian imputation, and variables were formatted prior to the development of the prediction tool.

Dietary sodium intake was the primary outcome (i.e., the variable to be predicted). Twenty-four-hour urinary sodium excretion was used as the reference standard for estimating dietary sodium intake. The completeness of the urine collection was assessed using urinary creatinine excretion (Normal range from 6 to 14 mmol/24 h in women and 8 to 16 mmol/24 h in men) and confirmed through patient interviews. Twenty-four-hour natriuresis was discretized into four clinically relevant classes: less than 5 g/day of salt, between 5 and 7 g/day of salt, between 7 and 9 g/day of salt, and more than 9 g/day of salt.

### 2.4. Dietary Self-Questionnaire

The “UniverSel” questionnaire [15] was developed with the assistance of a registered dietitian to estimate dietary sodium intake. This self-administered tool, adapted from an instrument originally developed by Dr. Robard Martin [16,17], collects information on:

(i) the frequency of consumption of the following foods: bread, sandwich bread, pastries, cakes, breakfast cereals and/or biscuits, salted butter, cheese, processed meats, canned or smoked fish (e.g., tuna, sardines, smoked salmon, anchovies, mussels, or salted cod), pizzas, French fries, fast food and/or sandwiches, chips and/or salty snacks, ready-made meals, canned foods and/or industrial soups, salt added to cooking water, salt added at the table, meat or vegetable stock, commercial sauces, restaurant meals, and commercial carbonated mineral water;
(ii) portion size, self-assessed by patients in comparison to peers of the same age, categorized as “less than,” “more than,” or “about the same as” peers.

### 2.5. Prediction Model—Bayesian Network

The sodium intake estimation tool was developed using a Tree-Augmented Naive Bayes (TAN) model, a type of directed acyclic probabilistic graphical model. In such networks, nodes represent variables, while directed arcs reflect conditional probabilistic dependencies, in accordance with Bayes’ theorem. These models enable the integration of both prior knowledge derived from clinical expertise and statistical relationships learned from empirical data.

The TAN model features a hierarchical structure based on three principles:

(i) each explanatory variable is directly connected to the target variable (i.e., 24-h urinary sodium excretion);
(ii) each explanatory variable may also be connected to one other explanatory variable, through a single parent node;
(iii) the final structure is the one that maximizes the total mutual information between the explanatory variables and the target, thereby optimizing predictive performance without increasing model complexity.

The model’s conditional parameters were estimated using the Expectation-Maximization (EM) algorithm, an iterative procedure that computes maximum likelihood estimates even in the presence of missing or latent data.

The influence of each explanatory variable on the target variable was quantified using belief variance reduction, an indicator derived from Bayesian theory that estimates each variable’s relative contribution to prediction. A sensitivity analysis was subsequently conducted to rank the predictors according to their relative importance. To enhance the model’s practicality while preserving its predictive performance, the number of variables was arbitrarily limited to the 15 most informative predictors based on belief variance reduction.

An initial internal validation, based on the “test with cases” approach, was conducted on the training dataset. This procedure involves comparing model predictions to known cases from the dataset used for model learning, in order to verify the consistency of the modeled conditional relationships and identify any structural inconsistencies.

The final model’s performance was assessed using accuracy, defined as the proportion of correct predictions among all analyzed cases, given that the target variable is categorical (comprising four levels of sodium intake).

All steps of model construction, training, and visualization were performed using Netica^®^ software (version 5.19, Norsys Corporation^®^, 3512 West 23rd Avenue, Vancouver, BC V6S 1K5, Canada).

### 2.6. Statistical Analysis

For each quantitative variable, the mean ± standard deviation (SD) was calculated when the distribution was normal; otherwise, data were presented as median [interquartile range]. All variables were formatted to be categorical for the purpose of model development. Continuous variables were automatically discretized into clinically meaningful classes based on frequency distribution.

### 2.7. Ethics Statement

Pseudonymized data were collected, and all procedures strictly adhered to the ethical principles outlined in the Declaration of Helsinki for medical research involving human subjects. The SUD-Méditerranée I ethics committee’s approval was obtained on 12 April 2023 under reference 023-A00590-45. In accordance with the French Jardé law, this cross-sectional study conducted within the framework of routine care that did not involve any additional examination, written information was provided to all subjects, and oral informed consent was mandatory and obtained from all participants prior to inclusion in the study. The study was registered at ClinicalTrials.gov under the identifier NCT05783960.

## 3. Results

### 3.1. Study Population

Among the 1089 patients initially selected to participate in the UniverSel study, 244 were excluded for the following reasons: significant extrarenal losses (*n* = 85 due to excessive sweating, intense physical activity, vomiting, diarrhea, or breastfeeding); unreliable urinary excretion (*n* = 57); absence of the UniverSel dietary questionnaire (*n* = 10); use of treatments likely to interfere with urinary sodium excretion (*n* = 46); specific clinical profile not representative of patients with CKD or with genetically determined specific sodium renal handling (*n* = 46), including Gitelman syndrome, Bartter syndrome, anorexia nervosa, or primary hyperaldosteronism.

**Figure 1:**
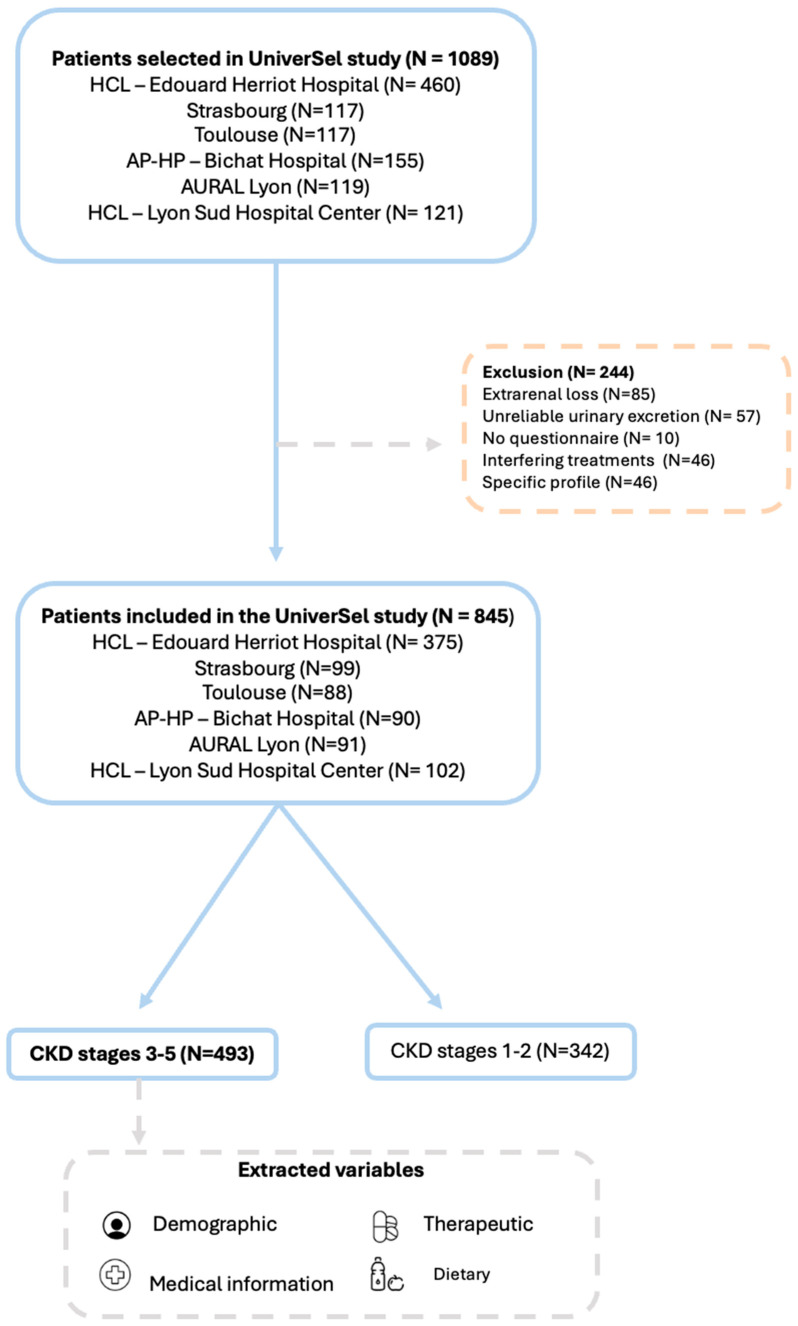
Flowchart of the study population showing patients selected, analyzed, and excluded from the study.

The learning database was subsequently divided into two groups according to CKD stages: CKD stage 1 and 2 and CKD stage 3 to 5 (eDFG < 60 mL/min/1.73 m^2^). The sodium intake estimation tool was developed using data from the 493 stage 3 to 5 CKD patients followed in one of the six participating French centers of the UniverSel study. None of the patients refused to participate (Figure 1).

The learning database, consisting of these 493 patients, included 36% women, with a mean age of 70 ± 11 years, a mean height of 167 ± 9 cm, and a mean weight of 77 ± 15 kg. The mean estimated glomerular filtration rate (eGFR), calculated using the CKD-EPI equation, was 39 ± 12 mL/min/1.73 m^2^.

The mean 24-h urinary creatinine excretion was 10.9 ± 3.8 mmol/24 h. Sodium excretion over 24 h was categorized into four groups: 24.9% of patients had an intake below 5 g/day, 26.6% between 5 and 7 g/day, 23.3% between 7 and 9 g/day, and 25.2% above 9 g/day. Vascular nephropathy was the most frequent etiology (37.1%), followed by diabetes (18.1%) and tubulointerstitial nephropathy (12.6%). All population characteristics are detailed in Table 1.

No predictive variable had more than 3% missing data at any time, and most had less than 1%. Missing data among predictive variables were imputed using a Bayesian network model, which generates plausible values from the posterior predictive distribution conditional on the observed data.

A center-specific description of the population used to develop the tool is available in the Supplementary Materials (Table S2).

### 3.2. Variables Selected for the Development of the Sodium Intake Estimation Tool and Internal Validation to Estimate Sodium Intake

In a first step, the 25 variables collected in the UniverSel study were ranked according to their contribution to belief variance using a Bayesian network approach (Table 2). This analysis aimed to identify the most informative predictors of 24-h urinary sodium excretion while limiting the number of input variables to ensure the tool’s practicality and ease of use in routine clinical settings. A balance between predictive performance and usability was sought.

The 15 variables most strongly associated with the target variable—24-h urinary sodium excretion, stratified into four classes—were selected for the development of the sodium intake estimation tool for patients with chronic kidney disease (CKD) stage 3–5. The variables, ranked in descending order of belief variance contribution, were: weight, height, sex, frequency of bread consumption, type of nephropathy, subjectively assessed food portion size, frequency of breakfast pastry consumption, age, frequency of consumption of commercial sauces, salted sparkling water, SBP, frequency of consumption of chips and/or appetizer snacks, pastries, industrial foods (e.g., pizza or fast food), and industrial cold cuts. All 25 analyzed variables are listed in descending order of belief variance in Table 2.

**Figure 2:**
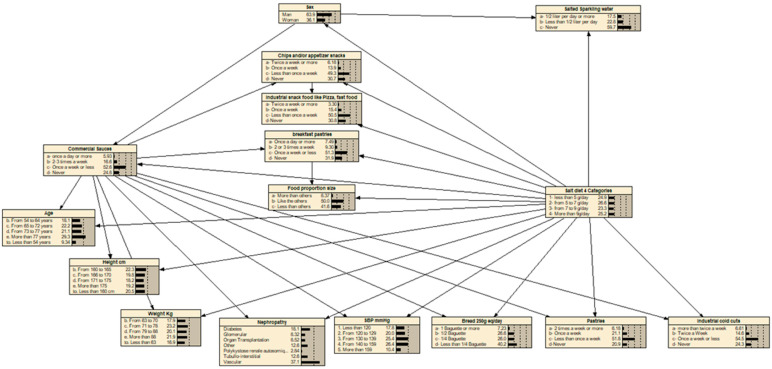
Optimized Bayesian network structure of the tool for estimating sodium consumption. *The values for each modality of each variable represent the distribution expressed in percentage.* **Abbreviation:** Nephropathy—nature of the nephropathy; SBP—Systolic Blood Pressure.

In a second step, an optimized Bayesian network model was developed using only these 15 informative variables. The structure of the final Bayesian network used for sodium intake prediction is shown in Figure 2.

**Figure 3:**
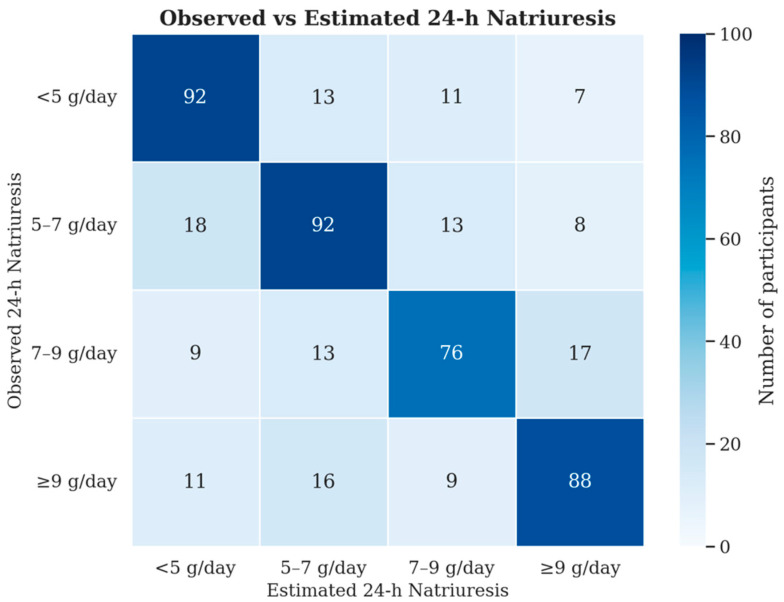
Confusion matrix comparing observed and predicted 24-h urinary sodium excretion classes for the 493 patients analyzed in the UniverSel study.

Finally, in a third step, the predictive performance of the optimized model was evaluated through internal validation using a test-with-case approach, comparing the predicted and observed classes of 24-h urinary sodium excretion in the validation dataset. The results, presented in Figure 3, demonstrate an overall prediction accuracy of 71%.

Figure 3 shows the agreement between observed and predicted 24-h urinary 24-h urinary sodium excretion (natriuresis) stratified into four intake categories: <5 g/day, 5–7 g/day, 7–9 g/day, and >9 g/day. Values represent the number of patients in each prediction-observation pair. Diagonal values indicate correctly classified cases. The overall prediction accuracy was 71%.

## 4. Discussion

The second UniverSel study aimed to develop a tool for estimating sodium and potassium intake using data collected from six specialized centers in nephrology, endocrinology, and cardiology. This paper presents the results of the analysis focused on estimating dietary sodium intake in patients with stage 3 to 5 CKD.

The development of this tool will serve a dual purpose. On the one hand, it will enable healthcare professionals to deliver more personalized care by tailoring dietary advice and treatment adjustments during clinical visits, with the goal of improving patient quality of life and reducing cardiovascular risk. On the other hand, it will empower patients at home by raising awareness of their nutritional intake and encouraging self-regulation of sodium consumption, thus enhancing prevention.

The sodium diet estimation tool is based on a Bayesian network model, a machine learning algorithm capable of autonomously learning from a training dataset. The Bayesian model, grounded in probabilistic reasoning, emulates clinical decision-making by estimating the likelihood of a disease given specific demographic factors, clinical signs, radiological findings, and biological results. It is particularly well-suited to the medical field as it incorporates both expert prior knowledge and empirical data. Furthermore, Bayesian networks offer several major advantages: they handle uncertainty in variable relationships, manage missing data and collinearity—frequent in health data—and are free from strong a priori assumptions that could introduce bias. To our knowledge, this is the first application of such a model to estimate dietary intake. Most existing predictive tools rely on linear regression models, which are poorly equipped to account for the complex, nonlinear interactions often encountered in medical data. The model used here, a Tree Augmented Naive Bayes network, captures inter-variable relationships without overwhelming computational complexity, improving accuracy while remaining fast and efficient. However, Bayesian models reveal probabilistic associations that may reflect causal relationships as well as coincidental ones. Therefore, careful interpretation and appropriate commentary are essential to help readers place these results in the proper context.

The model was developed using 15 explanatory variables: weight, height, sex, frequency of bread consumption, type of nephropathy, subjectively assessed portion size, frequency of breakfast pastry consumption, age, frequency of consumption of commercial sauces, salted sparkling water, SBP, frequency of consumption of chips and/or appetizer snacks, pastries, industrial foods (e.g., pizza or fast food), and industrial cold cuts. The target variable (sodium intake) was discretized into four categories to balance statistical robustness with clinical relevance: Less than 5 g/day, Between 5 and 7.5 g/day, Between 7.5 and 10 g/day and More than 10 g/day.

Surprisingly, the three variables most strongly associated with sodium intake were physical characteristics—weight, height, and sex—rather than dietary habits as initially expected. These three predictors (weight, height, sex) are objective and unlikely to be misreported, whereas dietary surveys often under- or overestimate intake. Patients with CKD routinely receive personalized dietary counseling through regular consultations and structured therapeutic education programs. As a result, their dietary habits are strongly influenced by professional guidance. In our study, the average salt intake among CKD patients was markedly lower (7.2 ± 2.9 mmol/day) than that typically observed in European populations (approximately 10 g/day). Nevertheless, across all four categories of salt intake, clear relationships persisted with body weight, height and, consistently, with overall caloric intake as well. In the Bayesian network analysis, weight and height were examined separately in relation to BMI. Since the associations were largely comparable, we retained the directly measured variables—weight and height—as separate predictors instead of BMI. A body weight greater than 88 kg was associated with a mean daily salt intake of 9.8 ± 4.5 g/day, whereas patients weighing less than 63 kg consumed an average of 5.8 ± 3.8 g/day. Height was also positively associated with salt intake (>175 cm: 9.5 ± 4.5 g/day vs. <160 cm: 6.2 ± 3.7 g/day). Age showed an inverse relationship (>77 years: 7.0 ± 3.9 g/day vs. <59 years: 9.0 ± 4.4 g/day). Female patients reported lower salt intake (6.4 ± 4.0 g/day) than male patients (8.6 ± 4.4 g/day). Interestingly, the item "Portion size", subjectively assessed using the questionnaire developed by Dr. Robard-Martin [16,17] which asks whether the patient believes they eat “more”, “less”, or “about the same” as those around them—was retained in our model. Overall, perceived dietary behaviors, with individuals who reported “eating more than others” exhibiting higher salt intake (9.2 ± 4.7 g/day) compared with those who reported “eating less than others” (6.8 ± 4.1 g/day), may reflect individual characteristics like weight, height, gender and age.

Systolic blood pressure demonstrated a modest positive association with sodium intake (SBP >159 mmHg: 7.7 ± 4.6 g/day vs. SBP <120 mmHg: 7.5 ± 4.4 g/day), consistent with the results of our dose–response meta-analysis conducted in patients with CKD [18]. Regarding CKD etiology, diabetic nephropathy was most strongly associated with higher sodium intake (8.6 ± 4.6 g/day), whereas autosomal dominant polycystic kidney disease was associated with lower sodium intake (6.8 ± 3.7 g/day). Other relevant predictors included SBP and the type of nephropathy. Interestingly, long-term diuretic therapy frequently prescribed to CKD patients does not interfere with urinary sodium excretion, making our tool usable in them.

Among our patients, the foods most strongly associated with sodium intake, in decreasing order, were: bread, breakfast pastries, commercial sauces, salted sparkling water, chips and/or appetizer snacks, pastries, industrial foods (e.g., pizza or fast food), and industrial cold cuts. Bread consumption was the most discriminant factor in France, with intake exceeding 250 g/day associated with 9.5 ± 4.3 g/day of salt versus 7.0 ± 4.2 g/day among those consuming less than 50 g/day.

The tool was developed using a multicenter database including 493 patients followed across six French centers, representing a wide range of clinical profiles—from mild to severe—thus enhancing the representativeness of the French CKD population. The model demonstrated satisfactory internal performance, with an accuracy of 71%, meaning that predicted and observed sodium intake categories matched in 71% of cases. This just exceeds the commonly accepted threshold of 70% for a tool to be considered clinically reliable. Several strategies could be considered to further improve predictive accuracy beyond the 70% threshold observed in our study. First, training the model on a larger dataset would likely enhance its generalizability and robustness. Second, collecting dietary intake data with more accurate methods than non-specific food frequency questionnaires, which only capture habitual frequencies, may reduce misclassification. Third, the use of repeated 24-h urinary collections (three times) would provide a more reliable estimate of dietary sodium intake and reduce measurement error. Finally, the application of more advanced machine learning approaches, such as ensemble methods or deep learning, could help capture complex and non-linear relationships between predictors and sodium intake. Together, these strategies may substantially improve model performance in future research.

In the medium term, this tool is intended to help reduce cardiovascular morbidity and mortality through increasingly personalized care, and to delay the need for renal replacement therapy. To facilitate its integration into everyday life in the digital age, we plan to make the tool available online via a website and through existing health applications—such as remote monitoring or prevention apps—accessible via smartphone, tablet, or computer.

However, this study has certain limitations. The main limitation lies in the use of 24-h urinary sodium excretion as the reference standard for estimating sodium intake. Although commonly used, this method has known reliability issues. To mitigate potential bias, patients—most of whom have been followed for many years—were thoroughly instructed on urine collection procedures. In cases where sample adequacy was questionable (assessed via 24-h urinary creatinine), physicians re-interviewed the patient regarding collection methods. Overall, 24-h urinary creatinine values in this study were satisfactory. It is important to note that no repeated collections were performed, and that 3-day urinary sodium collection remains the gold standard for estimating average sodium intake.

The main methodological limitation of this study is the lack of external validation. Before implementation in clinical practice, it is essential to assess the tool’s performance using an independent external dataset—national or international—to confirm its robustness and generalizability.

## 5. Conclusions

This study led to the development of an innovative clinical tool, based on an artificial intelligence model, capable of reliably estimating sodium intake in patients with chronic kidney disease stages 3 to 5. Tested using all the learning dataset, internal validation of the model demonstrated an overall accuracy of 71%. The tool was developed using the 15 most informative variables: weight, height, sex, frequency of bread consumption, type of nephropathy, subjectively assessed food portion size, frequency of breakfast pastry consumption, age, frequency of consumption of commercial sauces, salted sparkling water, SBP, frequency of consumption of chips and/or appetizer snacks, pastries, industrial foods (e.g., pizza or fast food), and industrial cold cuts.

In the medium term, this tool could contribute to more personalized patient management. Its future integration into digital platforms accessible in daily life—websites, health applications, and remote monitoring tools—represents a promising perspective to support its clinical use. However, before implementation in routine practice, external validation using national or international datasets will be essential to ensure robustness and generalizability. For research purposes, readers may access the salt consumption estimation tool via the following link: UniverSel-Salt (https://dev.hed.cc/?a=UniverSel&n=2025%20Reseau%20B%20creation%203%20Salt%20IRC%2015v%204%20Cat%20autodiscretize%20(10).neta, accessed on 16 October 2025).

## Figures and Tables

**Table 1:** Baseline characteristics of CKD patients used for the development of the sodium intake estimation tool—all centers combined (UniverSel study).

	All Centers		
**Number of Patients**			**493**
**Sodium Intake**			**Percentage (%)**
Less than 5 g/day			24.9
5 to 6.9 g/day			26.6
7 to 9 g/day			23.3
More than 9 g/day			25.2
**Patient Characteristics**	**Mean**	**SD**	**Percentage (%)**
Gender			
M			64
F			36
Age (years)	70	11	
a. Less than 54			9.3
b. 54 to 64			18.1
c. 65 to 72			22.1
d. 73 to 77			21.1
e. More than 77			29.2
Weight (kg)	77	15	
a. Less than 63			20.1
b. 63 to 70			21.7
c. 71 to 78			19.3
d. 79 to 88			17.8
e. More than 88			18.9
Height (cm)	167	9	
a. Less than 160			16.8
b. 160 to 165			17.8
c. 166 to 170			23.1
d. 171 to 175			20.1
e. More than 175			21.9
Nephropathy			
Vascular			37.1
Diabetes			18.1
Tubulointerstitial			12.6
Other			12.4
Glomerular			8.3
Organ transplantation (liver, heart, pulmonary)			8.2
Autosomic dominant polycystic kidney disease			2.8
Stage of CKD			
IIIa (45–59 mL/min)			36.9
IIIb (30–44 mL/min)			39.1
IV (15–29 mL/min)			20.9
V (<15 mL/min)			3.0
SBP (mmHg)	137	21	
1. Less than 120			17.7
2. 120 to 129			20.0
3. 130 to 139			25.4
4. 140 to 159			26.3
5. More than 159			10.4
DBP (mmHg)	73	12	
1. Less than 80			70.5
2. 80 to 84			11.5
3. 85 to 89			9.6
4. More than 89			8.2
Oedema (Yes)			3.6
Diabetes (Yes)			33.5
Heart failure			9.9
**Biology**	**Mean**	**SD**	**Percentage (%)**
CKD-EPI (ml/min/1.73 m^2^)	39	12	
24-h Diuresis (L/24 h)	2.0	0.7	
24-h Creatininuria (mmol/24 h)	10.9	3.8	
24-h Natriuresis (mmol/24 h)	123.5	49.5	
Plasma Bicarbonates (mmol/L)	24.5	3.2	
Plasma creatinine (µmol/L)	159.5	67.0	
Plasma sodium (mmol/L)	140	2.5	

**Abbreviation:** F—Female; M—Male; Nephropathy—nature of the nephropathy; SBP—Systolic Blood Pressure; DBP—Diastolic Blood Pressure; eGFR—estimated glomerular filtration rate.

**Table 2:** Ranking of the 25 baseline variables based on their contribution to belief variance in sodium intake estimation.

Variables		Percentage of Variance of Beliefs
	Variables included in the optimized Bayesian network	
1	Weight	4.94
2	Height	3.77
3	Sex	3.03
4	Bread consumption	2.22
5	Nephropathy	2.18
6	Food Portion Size	2.01
7	Breakfast pastries consumption	1.87
8	Age	1.85
9	Commercial sauces consumption	1.08
10	Salted sparkling water consumption	0.998
11	Systolic blood pressure	0.954
12	Chips and/or appetizer snacks consumption	0.947
13	Pastries consumption	0.893
14	Industrial snack food like pizza, fast food consumption	0.842
15	Industrial cold cuts consumption	0.818
	Variables not included in the optimized Bayesian network	
16	Oedema	0.791
17	Mushrooms consumption	0.63
18	Kalemia	0.613
19	Heart Failure	0.599
20	Nephrotic syndrome	0.583
21	Chocolate consumption	0.574
22	Thiazides use	0.497
23	Furosemide use	0.472
24	Renin angiotensin system blockers use	0.457
25	Dry vegetables consumption	0

Abbreviation: eGFR—estimated glomerular filtration rate.

## Data Availability

All databases will be protected in a password-protected Excel file and stored on password-protected computers and stored in password-protected computers that are changed every 3 months. The databases will be destroyed in 20 years.

## Supplementary Materials

The following supporting information can be downloaded at: https://www.mdpi.com/article/10.3390/nu17213398/s1, Table S1: TRIPOD Checklist: Compliance Assessment for the Reporting of the Prediction Mod; Table S2: Baseline characteristics of CKD patients used for the development of the sodium intake estimation tool-by center (UniverSel study); Table S3: UniverSel Food Questionnaire.

## Abbreviations

ADPKD	Autosomal dominant polycystic kidney disease
CKD	Chronic Kidney Disease
DBP	Diastolic Blood Pressure
eGFR	Estimation glomerular Filtration
EM	Expectation-Maximization
FFQ	Food frequency questionnaire
HTN	Hypertension
SBP	Systolic Blood Pressure
SD	Standard Deviation
TAN	Tree-Augmented Naive Bayes
