# Supplementary material for: AI-BASED Tool to Estimate Sodium Intake in STAGE 3 to 5 CKD Patients—The UniverSel Study
Source: Nutrients. 2025 Oct 29;17(21):3398. doi: 10.3390/nu17213398 (PMC12610316; doi:10.3390/nu17213398)
Supplement: Supplementary file 1 [file nutrients-17-03398-s001.zip › nutrients-3854166-supplementary.pdf]

nutrients

MDPI

Supplementary data

PREDICTION TOOL TO ESTIMATE SODIUM DIET IN STAGE 3 TO 5 CKD PATIENTS DEVELOPPED USING A MACHINE LEARNING TOOL – The UniverSel Study

Performance of an AI prediction tool to estimate sodium diet in CKD patients – The UniverSel Study

Section/Topic	m	Checklist Item	Page
**Title and abstract**			
Title	1	Identify the study as developing and/or validating a multivariable prediction model, the target population, and the outcome to be predicted.	1
Abstract	2	Provide a summary of objectives, study design, setting, participants, sample size, predictors, outcome, statistical analysis, results, and conclusions.	1
**Introduction**			
Background and objectives	3a	Explain the medical context (including whether diagnostic or prognostic) and rationale for developing or validating the multivariable prediction model, including references to existing models.	2
	3b	Specify the objectives, including whether the study describes the development or validation of the model or both.	2 - 3
**Methods**			
Source of data	4a	Describe the study design or source of data (e.g., randomized trial, cohort, or registry data), separately for the development and validation data sets, if applicable.	3
	4b	Specify the key study dates, including start of accrual; end of accrual; and, if applicable, end of follow-up.	3
Participants	5a	Specify key elements of the study setting (e.g., primary care, secondary care, general population) including number and location of centres.	3
	5b	Describe eligibility criteria for participants.	3
	5c	Give details of treatments received, if relevant.	NA
Outcome	6a	Clearly define the outcome that is predicted by the prediction model, including how and when assessed.	3 - 4
	6b	Report any actions to blind assessment of the outcome to be predicted.	NA
Predictors	7a	Clearly define all predictors used in developing or validating the multivariable prediction model, including how and when they were measured.	3 - 4
	7b	Report any actions to blind assessment of predictors for the outcome and other predictors.	NA

Sample size	8	Explain how the study size was arrived at.	NA
Missing data	9	Describe how missing data were handled (e.g., complete-case analysis, single imputation, multiple imputation) with details of any imputation method.	5
Statistical analysis methods	10a	Describe how predictors were handled in the analyses.	4-5
	10b	Specify type of model, all model-building procedures (including any predictor selection), and method for internal validation.	5
	10d	Specify all measures used to assess model performance and, if relevant, to compare multiple models.	5
Risk groups	11	Provide details on how risk groups were created, if done.	5
**Results**			
Participants	13a	Describe the flow of participants through the study, including the number of participants with and without the outcome and, if applicable, a summary of the follow-up time. A diagram may be helpful.	6-7
	13b	Describe the characteristics of the participants (basic demographics, clinical features, available predictors), including the number of participants with missing data for predictors and outcome.	7-8-9-10
Model development	14a	Specify the number of participants and outcome events in each analysis.	11
	14b	If done, report the unadjusted association between each candidate predictor and outcome.	11
Model specification	15a	Present the full prediction model to allow predictions for individuals (i.e., all regression coefficients, and model intercept or baseline survival at a given time point).	12
	15b	Explain how to the use the prediction model.	12
Model performance	16	Report performance measures (with CIs) for the prediction model.	12
**Discussion**			
Limitations	18	Discuss any limitations of the study (such as nonrepresentative sample, few events per predictor, missing data).	13
Interpretation	19b	Give an overall interpretation of the results, considering objectives, limitations, and results from similar studies, and other relevant evidence.	13
Implications	20	Discuss the potential clinical use of the model and implications for future research.	13
**Other information**			
Supplementary information	21	Provide information about the availability of supplementary resources, such as study protocol, Web calculator, and data sets.	Suppl. Data
Funding	22	Give the source of funding and the role of the funders for the present study.	14

Table S1. TRIPOD Checklist: Compliance Assessment for the Reporting of the Prediction Mod

	HEH			APHP			AURAL			CHLS			Strasbourg			Toulouse		
**Number of patients**			**242**			**22**			**62**			**71**			**67**			**29**
**Sodium intake**			**Percentage (%)**			**Percentage (%)**			**Percentage (%)**			**Percentage (%)**			**Percentage (%)**			**Percentage (%)**
Less than 5g/day			24.4			31.8			29.0			23.9			17.9			34.5
5 to 6.9g/day			26.4			31.8			22.6			28.2			26.9			27,0
7 to 9g/day			25.2			13.6			24.2			21.1			22.4			20.7
More than 9g/day			24.0			22.7			24.2			26.8			32.8			17.2
**Patient characteristics**	**Mean**	**SD**	**Percentage (%)**	**Mean**	**SD**	**Percentage (%)**	**Mean**	**SD**	**Percentage (%)**	**Mean**	**SD**	**Percentage (%)**	**Mean**	**SD**	**Percentage (%)**	**Mean**	**SD**	**Percentage (%)**
Gender																		
M			61.4			72.7			41.9			76.1			47.8			44.8
F			38.6			27.3			58.1			23.9			52.2			55.2
Age (years)	69.6	11.7		52.3	11.9		72.1	10.5		72.8	10.7		68.5	11.3		68.5	11.2	
a. Less than 54			11.2			40.9			4.8			7.0			11.9			10.3
b. 54 to 64			18.8			40.9			14.5			12.7			16.4			31.0
c. 65 to 72			22.8			4.5			29			21.1			28.3			13.8
d. 73 to 77			19.2			4.5			17.7			19.7			23.9			20.7
e. More than 77			28			9.1			32.2			39.4			19.4			24.1
Weight (kg)	77.0	13.0		76.2	14.9		77	13		76.6	15.3		79.7	15.5		72.1	17.1	
a. Less than 63			19.2			18.2			14.5			16.9			17.9			37.9
b. 63 to 70			14			18.2			16.4			14.1			8.7			17.2
c. 71 to 78			24.4			36.4			19.3			32.4			22.4			10.3
d. 79 to 88			18.8			18.2			24.6			11.3			23.9			13.8

e. More than 88			23.6			9.1			24.6			25.3			26.9			20.7
Height (cm)	167.1	9.3		169,7	9.9		166.0	9.2		167.2	8.9		168.1	9.2		164.8	9.6	
a. Less than 160			18.8			9.1			21.4			16.9			14.5			34.5
b. 160 to 165			26.7			31.8			30.3			22.5			30.6			17.2
c. 166 to 170			20.4			22.7			16.1			29.6			14.5			17.2
d. 171 to 175			13.3			0			17.9			14.1			14.5			10.3
e. More than 175			20.8			36.4			14.3			16.9			25.8			20.7
Nephropathy																		
Vascular			29.1			27.3			37.7			25.3			13.4			58.6
Diabetes			21.9			4.5			21.3			22.5			29.8			17.2
Tubulointerstitial			13.1			27.3			11.5			18.3			8.9			3.4
Other			13.5			16.2			16.4			11.3			13.4			10.3
Glomerular			11.1			16.2			3.3			14.1			17.9			0
Organ transplantation (liver, heart, pulmonary)			9.2			0,0			6.6			7.0			16.4			10.3
Autosomic dominant polycystic kidney disease			1.6			4.5			3.3			1.4			0,0			0
Stage of CKD																		
IIIa (45-59 ml/min)			38.2			54.5			27.4			36.6			37.3			55.2
IIIb (30-44 ml/min)			39,0			40.9			37.1			42.2			40.3			31.0
IV (15-29 ml/min)			17.9			4.5			22.6			19.7			19.4			10.3
V (< 15 ml/min)			4.8			0,0			12.9			1.4			3.0			3.4
CKDEPI	39	12		45	9		34	14		39.4	11.3		38.2	11.3		44.2	11.7	
SBP (mmHg)	140	2		132	10		141	24		141.2	15.7		131.7	12.3		157.3	38.0	
1. Less than 120			10.9			14.3			9.1			5.6			12.7			20.7

2. 120 to 129			20.1		23.8	18,9			21.8			16.9			27.0			6.9
3. 130 to 139			29.3		47.6	27,8			27.3			29.6			33.3			10.3
4. 140 to 159			27.6		14.3	23,3			27.3			35.2			25.4			24.1
5. More than 159			12.1		0	5,5			14.5			12.7			1.6			37.9
DBP (mmHg)	75	12.3		78	9		73	14		76.1	11.0		74.5	10.7		78.8	16.6	
1. Less than 80			62			57.1			66.7			62			63.5			51.7
2. 80 to 84			14.8			23.8			5.5			18.3			19.0			6.9
3. 85 to 89			11.4			14.3			16.7			8.5			6.3			17.2
4. More than 89			11.8			4.8			11.0			9.8			11.1			24.1
Oedema (Yes)			5.6			9.1			8.1			2.8			6.0			3.4
Diabetes (Yes)			38.2			31.8			35.5			33.8			46.3			41.4
Heart failure			6,7			4.5			11.3			16.9			7.5			3.4
**Biology**	**Mean**	**SD**		**Mean**	**SD**	**Percentage (%)**	**Mean**	**SD**		**Mean**	**SD**		**Mean**	**SD**		**Mean**	**SD**	**Percentage (%)**
CKD-EPI (ml/min/1.73m²)	39.4	11.3		45.4	9.5		33.8	14.1		39.4	11.3		38.2	11.3		44.2	11.7	
24-hour Diuresis (L/24h)	2.0	0.7		2.2	0.8		1.9	0.7		2.0	0.7		2.2	0.8		1.9	0.6	
24-hour Creatininuria (mmol/24h)	11	3.4		11.7	5.1		10	3.6		11.3	3.6		10.9	4.6		9.3	3.5	
24-hour Natriuresis (mmol/24h)	123.2	45.7		121.3	59.8		117.2	43.3		127.4	55.3		132.1	55.2		114.8	56.2	
Plasma Bicarbonates (mmol/L)	24.5	3.2		24.2	3.4		23.3	3.9		24.7	2.6		25.1	2.6		25.0	4.0	
Plasma creatinine (µmol/L)	157	58.1		146.1	36.8		196.3	114.3		154.7	50.4		154.3	52.9		136.3	60.9	
Plasma sodium (mmol/L)	139.3	2.6		139.7	1.6		139.5	2.9		139.6	2.0		139.5	2.5		140.6	2.3	

Table S2. Baseline characteristics of CKD patients used for the development of the sodium intake estimation tool - by center (UniverSel study).

Abbreviation: F- Female; M-Male; Nephropathy-nature of the nephropathy; SBP- Systolic Blood Pressure; DBP-Diastolic Blood Pressure; eGFR – estimated glomerular filtration rate

nutrients MDPI

Center number: |______| Patient number: |___|___|___|___|
First letter of last name | | First letter of first name |_ Date completed |___|___|___|___|___|___|___|

This questionnaire **UniverSel** is designed to assess your salt intake.

<u>You may sometimes hesitate when answering.</u> In this case, please tick the box that most closely matches your opinion.

Did you experience any of the following symptoms in the week prior to the urine collection:
Diarrhea: O Yes - O No, Vomiting: O Yes - O No, Sweating (exercise, fever): O Yes - O No,
Constipation (fewer than 3 bowel movements per week): O Yes - O No

<u>Day of urine collection</u> O Monday O Tuesday O Wednesday O Thursday O Friday O Saturday O Sunday |

1. Do you eat bread (equivalent to a 250g baguette)?
   - O 1 baguette per day
   - O 1/2 baguette per day
   - O 1/4 baguette per day
   - O Less than 1/4 baguette per day
2. Do you eat sandwich bread?
   - O 2 slices per day or more
   - O 1 slice per day
   - O Less than one slice per day
   - O Never
3. Do you eat pastries?
   - O 1 pastry per day or more
   - O 2-3 pastries per week
   - O 1 pastry per week or less
   - O Never
4. Do you eat cake?
   - O 2 cake per week or more
   - O 1 cake per week
   - O Less than one cake per week
   - O Never
5. Do you eat breakfast cereals and/or biscuits?
   - O Once or more per day
   - O 2-3 times per week
   - O Once a week or less
   - O Never
6. Do you eat salted butter?
   - O Regularly
   - O Rarely
   - O Never
7. Do you consume cheese, grated cheese, and/or cheese in prepared foods?
   - O 2 servings per day or more
   - O 1 serving per day
   - O Less than one serving per day
   - O Never
8. Do you consume sliced processed meats (including ham and chicken breast)?
   - O More than 2-3 times a week
   - O 2-3 times a week
   - O Once a week or less
   - O Never
9. Do you eat tuna, sardines, smoked salmon, anchovies, mussels, and/or salted cod?
   - O 2 times a week or more
   - O Once a week
   - O Less than once a week
   - O Never
10. Do you eat sandwiches, pizza, French fries, and/or fast food?
    - O Twice a week or more
    - O Once a week
    - O Less than once a week
    - O Never
11. Do you eat chips and/or salty snacks (including olives)?
    - O Twice a week or more
    - O Once a week
    - O Less than once a week
    - O Never
12. Do you eat ready-made meals, canned foods and/or industrial soups?
    - O Twice a week or more
    - O Once a week
    - O Less than once a week
    - O Never
13. Do you eat salt added to cooking water?
    - O Always
    - O Sometimes
    - O Rarely
    - O Never

Center number: I______I Patient number: I___I___I___I
First letter of last name I I First letter of first name I_ Date completed I___I___I___I___I___I___I

14. Do you eat salt added at the table?
   - O At every meal
   - O Once a day
   - O 2-3 times a week or less
   - O Never

15. Do you eat meat or vegetable stock?
   - O Twice a week or more
   - O Once a week
   - O Less than once a week
   - O Never

16. Do you eat commercial sauces (mayonnaise, mustard, industrial salad dressing, ketchup, Algerian and/or Asian sauce)?
   - O Once a day or more
   - O 2-3 times a week
   - O Once a week or less
   - O Never

17. Do you go to restaurants?
   - O 2-3 times a week or more
   - O Once a week
   - O Less than once a week
   - O Never

18. Do you drink sparkling mineral water (excluding Sodastream)?
   - O 0,5 liters per day or more
   - O Less than 0.5 liters per day
   - O Never

19. Do you eat (compared to someone of your age and gender in your circle of friends and family)?
   - o More than others
   - o Like others
   - o Less than others

Table S3. UniverSel Food Questionnaire
